# Large language models forecast patient health trajectories enabling digital twins

**DOI:** 10.1038/s41746-025-02004-3

**Published:** 2025-10-01

**Authors:** Nikita Makarov, Maria Bordukova, Papichaya Quengdaeng, Daniel Garger, Raul Rodriguez-Esteban, Fabian Schmich, Michael P. Menden

**Affiliations:** 1 Roche Innovation Center Munich (RICM), Penzberg, Germany; 2 Computational Health Center, Helmholtz Munich, Munich, Germany; 3 Department of Biology, Ludwig Maximilian University of Munich, Munich, Germany; 4 TUM School of Computation, Information and Technology, Technical University of Munich, Munich, Germany; 5 Roche Innovation Center Basel (RICB), Basel, Switzerland; 6 Department of Biochemistry and Pharmacology, Bio21 Molecular Science and Biotechnology Institute, The University of Melbourne, Melbourne, VIC Australia

**Keywords:** Computational biology and bioinformatics, Computational models, Data processing, Machine learning, Predictive medicine, Biomarkers, Outcomes research

## Abstract

Generative artificial intelligence is revolutionizing digital twin development, enabling virtual patient representations that predict health trajectories, with large language models (LLMs) showcasing untapped clinical forecasting potential. We developed the Digital Twin—Generative Pretrained Transformer (DT-GPT), extending LLM-based forecasting solutions to clinical trajectory prediction. DT-GPT leverages electronic health records without requiring data imputation or normalization and overcomes real-world data challenges such as missingness, noise, and limited sample sizes. Benchmarking on non-small cell lung cancer, intensive care unit, and Alzheimer’s disease datasets, DT-GPT outperformed state-of-the-art machine learning models, reducing the scaled mean absolute error by 3.4%, 1.3% and 1.8%, respectively. It maintained distributions and cross-correlations of clinical variables, and demonstrated explainability through a human-interpretable interface. Additionally, DT-GPT’s ability to perform zero-shot forecasting highlights potential advantages of LLMs as clinical forecasting platforms, proposing a path towards digital twin applications in clinical trials, treatment selection, and adverse event mitigation.

## Introduction

Clinical forecasting involves predicting patient-specific health outcomes and clinical events over time, which is essential for patient monitoring, treatment selection, and drug development^1^. An emerging approach to support such forecasting is the use of digital twins^2,3^. These are virtual representations of patients that generate detailed, multivariable predictions of future health states by leveraging longitudinal medical history^3,4^. When initialized with individual patient characteristics, digital twins can simulate real-time personalized responses to medical interventions or treatments^2,4,5^.

Digital twins offer a comprehensive framework for patient modeling by integrating diverse data streams, which can include history of medical examinations, diagnoses and treatments, deep molecular profiling, lifestyle and environmental factors, as well as general biomedical knowledge^6–8^. They provide a holistic reflection of an individual’s status within the broader context of the patient population, accounting for the interplay of disease dynamics and medical interventions^4^. By bridging the gap between population-level evidence and individual-level insights, the application of digital twins is poised to revolutionize healthcare in areas such as precision and personalized medicine, predictive analytics, virtual testing, continuous monitoring, and enhanced decision support^3,4^.

Generative artificial intelligence (AI) holds promise for creating digital twins due to its potential to produce synthetic yet realistic data, but this area of application is still in its infancy^4^. Generative AI methods for predicting patient trajectories include recurrent neural networks, transformers and stable diffusion^9–13^. These often fall short in terms of handling missing data, interpretability and performance. These challenges can be partially addressed by causal machine learning^14^, but these algorithms face limitations related to small datasets or being confined to simulations^15^.

Recent breakthroughs in generative AI have been achieved with foundation models, which are pre-trained AI models adaptable to various specific tasks involving different types of data. Most foundation models for patient forecasting focus on single-point predictions rather than comprehensive longitudinal patient trajectories, which are needed for clinical decision-making^16^. Recently, clinically focused, LLM-inspired methods have been proposed^17^, however, with their evaluation focus still being on single-point predictions rather than longitudinal trajectories, and without using the knowledge of pretrained LLMs. Less explored for this purpose remain text-focused Large Language Models (LLMs), which have demonstrated forecasting capabilities^18,19^, including some approaches showing the ability of zero-shot forecasting, i.e., forecasting without any prior specific training in the task, thus highlighting their remarkable generalizability^20–22^.

LLM-based forecasting has made great progress in general forecasting. However, some common methods, such as LSTPrompt^20^, LLMTime^21^, Time-LLM^22^, and GPT4TS^23^, make assumptions which may not necessarily hold in clinical trajectory forecasting. One example is channel independence, whereby, for multivariate time series, channel-independent models process each time series separately, without modeling interactions and inter-time series dependencies. This approach may not be optimal in the clinical setting, in which we often observe correlated time series, putatively driven by causal biological links, highlighting the need to process all aspects of a patient simultaneously.

We propose the creation of digital twins based on LLMs that leverage data from electronic health records (EHRs) from real world data (RWD) and observational studies. EHRs are a key source of training data for machine learning models in healthcare, as they record patient characteristics such as demographics, diagnoses, and lab results over time^24^. However, they pose specific challenges such as data heterogeneity, rare events, sparsity, and quality issues^16^. There have been developments in machine learning to overcome these challenges, especially for data sparsity, usually by adapting the model’s architecture, resulting in increased model complexity and the introduction of further assumptions on the data^10,13^.

**Fig. 1:**
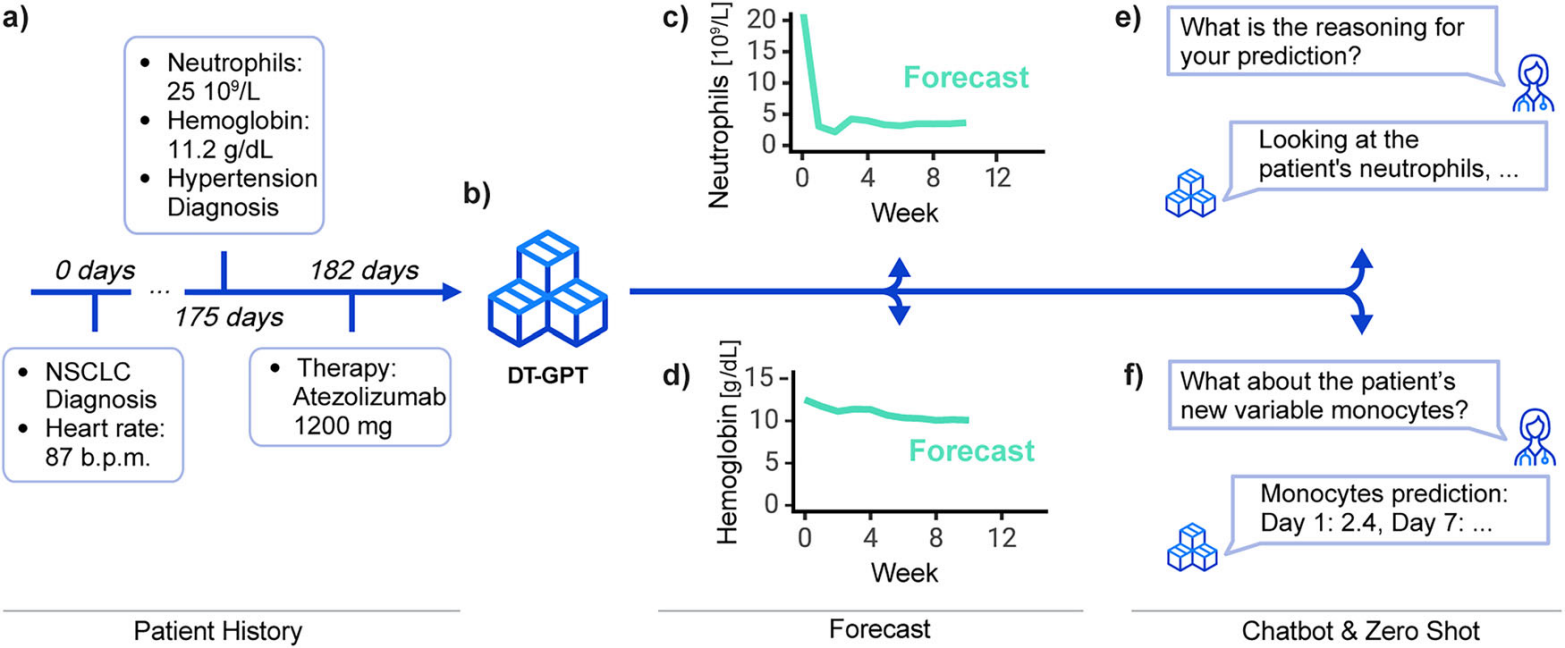
The LLM-based DT-GPT framework enables forecasting patient trajectories, identifying key variables, and zero-shot predictions. Here exemplified, **a** sparse patient timeline, which **b** DT-GPT utilizes for generating longitudinal clinical variable forecasts, e.g., **c** neutrophil and **d** hemoglobin blood levels. DT-GPT can **e** chat and respond to inquiries about important variables, as well as (**f**) perform zero-shot forecasting on clinical variables previously not used during training.

We hypothesize that LLMs will empower the next generation of digital twins in healthcare. Here, we introduce the Digital Twin - Generative Pretrained Transformer (DT-GPT) model (Fig. 1), which

enables: (i) forecasting of clinical variable trajectories, (ii) zero-shot predictions of clinical variables not previously trained on, and (iii) preliminary interpretability utilizing chatbot functionalities. DT-GPT is an extension of previous LLM-based forecasting solutions, based on fine-tuning LLMs on clinical data using a straightforward data encoding scheme. The method is designed to solve clinically specific issues, be model-agnostic and to be applied to any text-focused LLM without any further architectural changes.

## Results

We analyzed the performance of DT-GPT by forecasting various clinical values on diverse datasets, including on a short-term scale (next 24 h) for Intensive Care Unit (ICU) patients, a medium-term scale (up to 13 weeks) for non-small cell lung cancer (NSCLC) patients, as well as a long-term Alzheimer’s Disease dataset (next 24 months). The ICU dataset is based on Medical Information Mart for Intensive Care IV (MIMIC-IV)^25^ with 35,131 patients, whilst the NSCLC dataset is based on the the nationwide Flatiron Health EHR-derived de-identified database, containing 16,496 NSCLC patients (“Methods”; Supplementary Tables 1–4; Supplementary Note 1). The Alzheimer’s disease dataset is derived from the Alzheimer’s Disease Neuroimaging Initiative (ADNI) dataset, containing 1,140 patients (Supplementary Tables 1 and 5; Supplementary Note 1). The datasets complement the analysis to understand how the model works on short-, medium- and long-term scales, as well as on different amounts of patients available for training. All details on task setup, data preprocessing, model training, and evaluation are provided in the “Methods” section.

### DT-GPT achieved state-of-the-art forecasting performance

DT-GPT achieved the lowest overall scaled mean absolute error (MAE) across benchmark tasks in comparison with state-of-the-art models (Table 1), with the z-score scaling allowing comparison and aggregation across variables (“Methods”). In the NSCLC dataset, we predicted six laboratory values weekly for up to 13 weeks post-therapy initiation, leveraging all pre-treatment data to model patient trajectories under treatment. For the ICU task, we forecasted the next 24 h by predicting respiratory rate, magnesium and oxygen saturation based on the previous 24 h history, enabling real-time monitoring and timely intervention. In the Alzheimer’s dataset, we forecasted Mini Mental State Examination (MMSE)^26^, Clinical Dementia Rating sum of boxes (CDR-SB)^27^ and Alzheimer’s Disease Assessment Scale (ADAS11)^28^ cognitive scores, over the next 24 months at 6 month intervals using baselines measurements. All comparisons were performed on unseen patients.

**Table 1:** Benchmark of clinical variable forecasting across three datasets

Scaled mean absolute error	Model	Non-small cell lung cancer (NSCLC)						Intensive care unit			Alzheimer’s disease		
		Hemoglobin	Leukocytes	Lymphocytes/ Leukocytes	Lymphocytes	Neutrophils	Lactate Dehydrogenase	Magnesium	Resp. Rate	Oxygen Saturation	MMSE	CDR-SB	ADAS11
Channel-Independent Input	Copy Forward	0.698	0.969	0.731	0.569	0.974	0.433	0.681	0.769	0.746	0.654	0.539	0.519
	PatchTST	0.684	0.968	0.719	0.560	0.959	0.447	0.671	0.635	0.646	0.654	0.540	0.506
	Time-LLM	0.665	0.894	0.684	0.544	0.878	0.443	0.664	0.655	0.665	0.654	0.540	0.506
	LLMTime	0.736	0.923	0.725	0.601	0.900	0.437	0.759	0.686	0.688	0.654	0.539	0.503
Channel-Dependent Input	BioMistral-7B	0.984	1.097	0.756	0.997	1.953	0.600	0.790	0.770	0.945	2.064	0.883	0.728
	Qwen3-32B	0.670	0.942	0.736	0.573	0.937	0.453	0.709	0.720	0.791	0.686	0.546	0.555
	TCN	0.660	0.857	0.752	0.606	0.832	0.731	0.612	0.713	0.726	*Not Applicable*	*Not Applicable*	*Not Applicable*
	Linear Regression	0.486	0.782	0.668	0.506	0.778	0.475	0.606	0.680	0.681	0.551	0.449	**0.457**
	RNN	0.529	0.806	0.671	0.511	0.801	0.433	0.597	0.647	0.674	0.545	0.463	0.465
	Transformer	0.496	0.749	0.683	0.503	0.741	0.514	0.537	0.644	0.651	0.553	0.485	0.481
	LSTM	0.526	0.781	0.665	0.495	0.764	0.441	0.567	0.643	0.642	0.545	0.468	0.475
	Temporal Fusion Transformer	0.469	0.719	0.651	0.463	0.717	0.480	0.537	0.635	0.644	**0.520**	0.451	0.466
	TiDE	0.464	0.737	0.655	0.465	0.740	0.453	0.534	0.635	0.652	0.578	0.498	0.506
	LightGBM	0.453	0.727	0.644	0.456	0.734	0.425	0.520	**0.634**	0.644	0.540	0.455	0.462
	**DT-GPT (ours)**	**0.439**	**0.687**	**0.643**	**0.434**	**0.701**	**0.418**	**0.505**	0.636	**0.635**	0.535	**0.417**	0.458

DT-GPT outperformed the baselines in the majority of cases of the non-small cell lung cancer (NSCLC), intensive-care unit (ICU), and Alzheimer’s disease dataset. All errors refer to mean absolute error (MAE; lower is better) scaled by standard deviation.

We compared DT-GPT to 14 multi-step, multivariate baselines, ranging from a naïve model that copies over the last observed value to state-of-the-art forecasting models. These included linear regression model, time series LightGBM model, Temporal Fusion Transformer (TFT), Temporal Convolutional Network (TCN), Recurrent Neural Network (RNN), Long Short-Term Memory (LSTM), Transformer, and Time-series Dense Encoder (TiDE) model^12,29,30^. The naïve model ensured that models with better performance capture nonstationary time series, whilst advanced models were chosen for their ability to handle future variables and achieving state-of-the-art performance in both medical and standard time series forecasting^31,32^. To understand the contribution of fine-tuning, we also run the general, state-of-the-art LLM Qwen3-32B and the biomedical LLM BioMistral-7B^33,34^. Note that DT-GPT is a fine-tuned 7-billion-parameter model based on BioMistral, whilst Qwen3 is a significantly larger model at 32 billion parameters. Additionally, we benchmarked advanced time-series LLM-based methods, i.e. Time-LLM and LLMTime^21,22^, as well as a patch based model PatchTST^35^, all of which are channel-independent models, which process each input time series separately.

On the NSCLC dataset, DT-GPT achieved an average scaled MAE of 0.55 ± 0.04, whilst LightGBM, the second best model, achieved an average scaled MAE of 0.57 ± 0.05, showing a relative improvement of 3.4% (Table 1), On the ICU dataset, DT-GPT achieved an average scaled MAE of 0.59 ± 0.03, whilst the second best model, LightGBM, performed at 0.60 ± 0.03, equivalent to a 1.3% improvement (Table 1). On the Alzheimer’s disease dataset, DT-GPT achieved an average scaled MAE of 0.47 ± 0.03, with Temporal Fusion Transformer being the second best model with 0.48 ± 0.02, representing a relative improvement of 1.8%. We note that the scaled MAE is normalized by standard deviation, with DT-GPT consistently achieving absolute MAE (Supplementary Tables 6–17) that is lower than the standard deviation, indicating that forecasting errors are smaller than the natural variability present in the data. DT-GPT is shown to be the best performer out of 14 models across all datasets, and achieving statistical significance over the second-best performing model on the NSCLC (*p* value < 9.6162 × 10^−17^) and ICU (p-value < 0.00043) datasets (Supplementary Note 2; Supplementary Tables 18–19; Supplementary Figs. 1–2).

Channel-independent models, such as LLMTime, Time-LLM and PatchTST, perform worse with respect to scaled MAE on variables that are more sparse and correlate less with other time series. Inversely, we see that the channel-independent models perform relatively better on respiratory rate and oxygen saturation, which have generally more dense measurements and are less correlated to time series such as treatment, in comparison, for example, to neutrophils in NSCLC.

The LLMs without fine-tuning performed significantly worse than DT-GPT, often incorrectly hallucinating results. DT-GPT outperformed BioMistral by 47.9%, 29.1% and 61.1%, and outperformed the larger Qwen3-32B model by 22.9%, 19.9% and 21.1% on NSCLC, ICU and Alzheimer’s disease datasets, respectively.

To comprehensively evaluate DT-GPT, we assessed a range of metrics, including derived classification metrics (“Methods”). DT-GPT consistently performed well across various metrics, capturing trajectory trends effectively (Supplementary Tables 6–17). For forecasting, we compared scaled MAE, absolute MAE, mean absolute scaled error (MASE), symmetric mean absolute percentage error (SMAPE), and Spearman Correlation (“Methods”). For classification, we evaluated the area under the receiver operating characteristic curve (AUC) of clinically low/high predictions and overall trends (Supplementary Tables 6–11). These metrics collectively offer insights into different aspects of model performance (Supplementary Note 2).

DT-GPT shows strong potential in capturing clinically relevant lab trends but has limitations in predicting specific critical events. For example, DT-GPT struggled with forecasting critically low hemoglobin levels ( < 7.5 g/dL; ROC AUC = 0.506), likely due to their low prevalence (1.2%; Methods; Supplementary Tables 6–11; Supplementary Note 2). Similarly, prediction of high leukocyte counts ( > 11.0 × 10⁹/L) was modest (ROC AUC = 0.578) and fell below the copy-forward baseline (ROC AUC = 0.616).

Notably, DT-GPT demonstrated robust predictive performance across several routine yet clinically informative laboratory parameters. This includes detection of mild anemia (hemoglobin below reference; ROC AUC = 0.793) and elevated LDH (lactate dehydrogenase; >222 U/L; ROC AUC = 0.793), a marker of NSCLC progression^36^. It also captured three-week trends in hemoglobin (increasing/decreasing; ROC

AUCs = 0.704/0.638) and rising leukocytes, lymphocytes, and neutrophils suggestive of inflammation (ROC AUCs = 0.65–0.68)^37^.

**Fig. 2:**
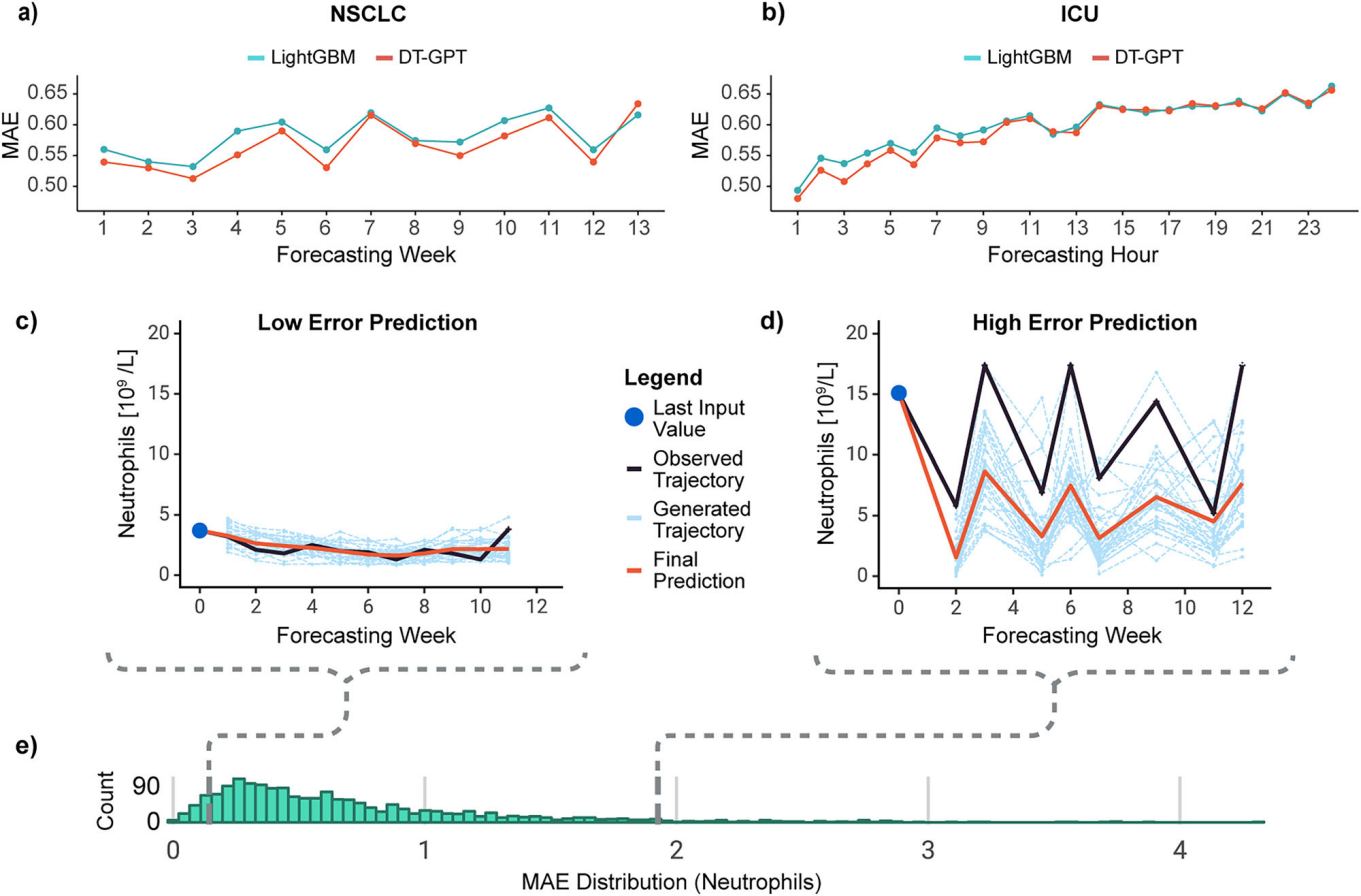
DT-GPT achieves state-of-the-art performance for clinical trajectory forecasting. **a** The long-term non-small cell lung cancer (NSCLC) and **b** the short-term intensive-care unit (ICU) dataset, with the x-axis showing relative time points and the y-axis the corresponding scaled mean absolute error (MAE), comparing with the second best forecasting model LightGBM. The scaling is done by the standard deviation, allowing comparison across variables with different value ranges and calculating a final performance score by averaging across the variables. Here exemplified, DT-GPT forecasts of neutrophil counts in patients with (**c**) low and **d** high error, for all weeks where the ground truth exists. **e** Histogram of MAE distribution for all predicted neutrophil counts.

DT-GPT forecasts preserved inter-variable relationships. The correlations between the variables forecasted by DT-GPT aligned with the correlations between the variables in the test datasets with an R^2^ of 0.98 and 0.99, whilst those of LightGBM achieved an R^2^ of 0.97 and 0.99 (Supplementary Fig. 3) on the NSCLC and ICU datasets, respectively. Additionally, DT-GPT outperformed LightGBM in the majority of timepoints in both datasets, demonstrating that the improvement was consistent across time (Fig. 2a, b). For Alzheimer’s disease, both DT-GPT and the second best model TFT achieved an R^2^ of 0.99.

DT-GPT can be further improved by utilising alternative trajectory aggregation methods. To inspect both low and high MAE predictions from DT-GPT, we visualized two sample individual-patient forecasts for the variable neutrophils (Fig. 2c, d) picked from the low and high end of performance distribution (Fig. 2e).

It is important to note that the final prediction was derived by averaging 30 generated trajectories and that, even in poor performing cases, individual non-averaged forecasted trajectories sometimes succeeded in capturing aspects of the true trajectory.

To assess the impact of trajectory aggregation, we calculated the error given an optimal aggregation. To this end, we selected the individual trajectories with the lowest scaled MAE and recalculated the hypothetical scaled MAE on the NSCLC dataset, achieving a 26% improvement in error to 0.40 ± 0.02, without any further model training, noting that this is a theoretical lower bound. Finally, we observed that in the distribution of scaled MAE for neutrophils across all patients, most of the errors were right-skewed, indicating that high errors came from a small number of patients with likely uncommon trajectories (Fig. 2e).

**Fig. 3:**
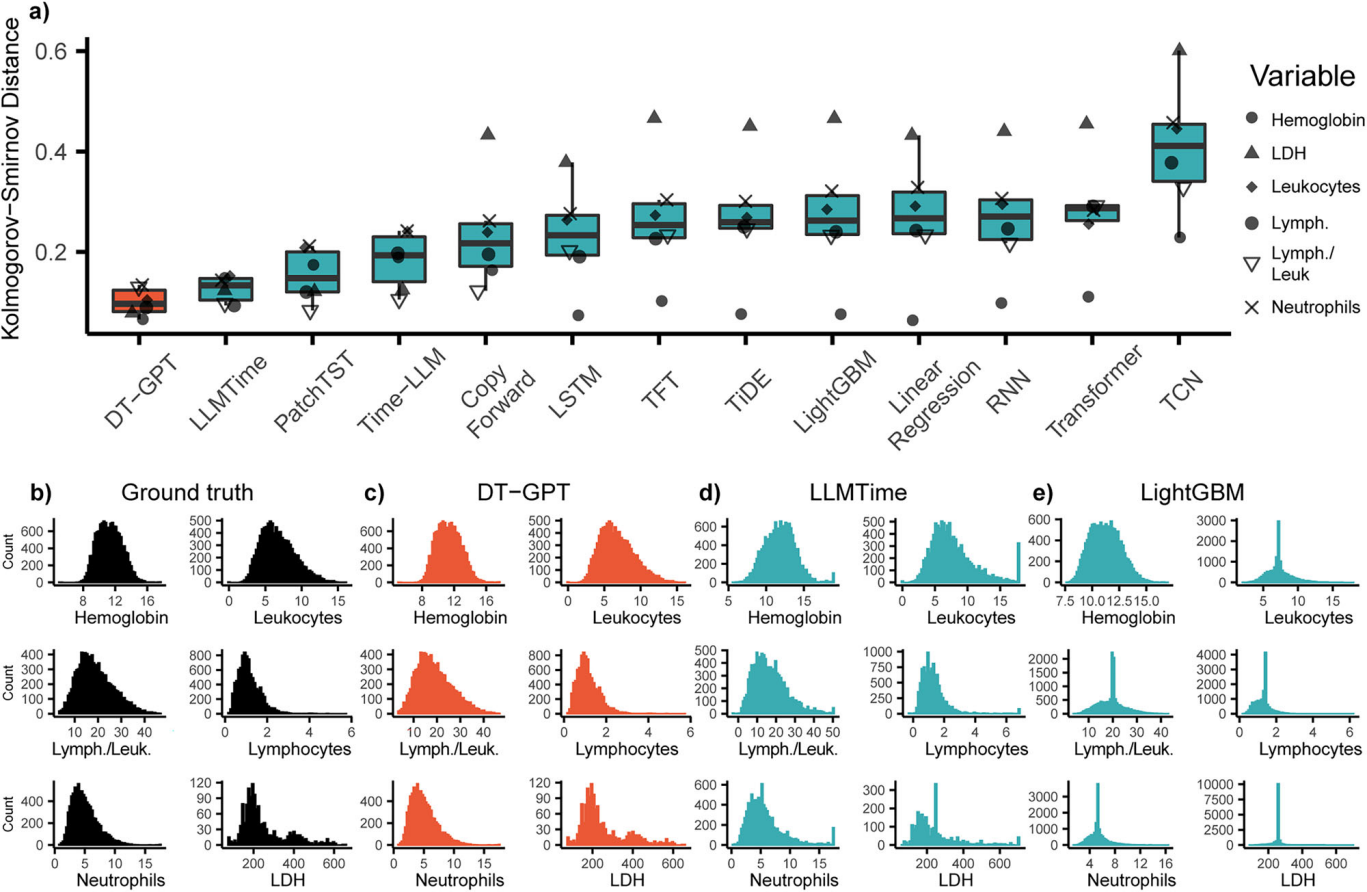
DT-GPT resembles the distribution of the original data. **a** The distribution of DT-GPT forecasted values is the closest to the ground truth distribution according to the absolute Kolmogorov-Smirnov distance. This can also be observed from the distribution histograms associated with (**b**) the ground truth, **c** DT-GPT, **d** LLMTime, and **e** LightGBM. While LightGBM has the lowest scaled MAE after DT-GPT, LLM-based methods such as LLMTime more accurately resemble ground-truth data distribution. Lymph./Leuk. lymphocytes/leukocytes).

DT-GPT preserves the overall distribution of target variables—a property that, while not sufficient, is arguably necessary for clinically meaningful forecasting. To assess this, we computed the Kolmogorov–Smirnov (KS) statistic across all target variables in the NSCLC cohort, comparing predicted and true distributions (Fig. 3a). DT-GPT exhibited the lowest median KS score among all models,

indicating the best distributional alignment. Notably, several recent baselines, including TiDE, TCN, and TFT, struggled with the distribution modeling. We also visualized the distributions of the ground truth (Fig. 3b) and DT-GPT predictions (Fig. 3c), alongside LLMTime which had the second lowest mean score on the Kolomogorov-Smirnov statistic (Fig. 3d), and LightGBM which was the best performing baseline with respect to scaled MAE (Fig. 3e).

### DT-GPT is robust to common RWD challenges

**Fig. 4:**
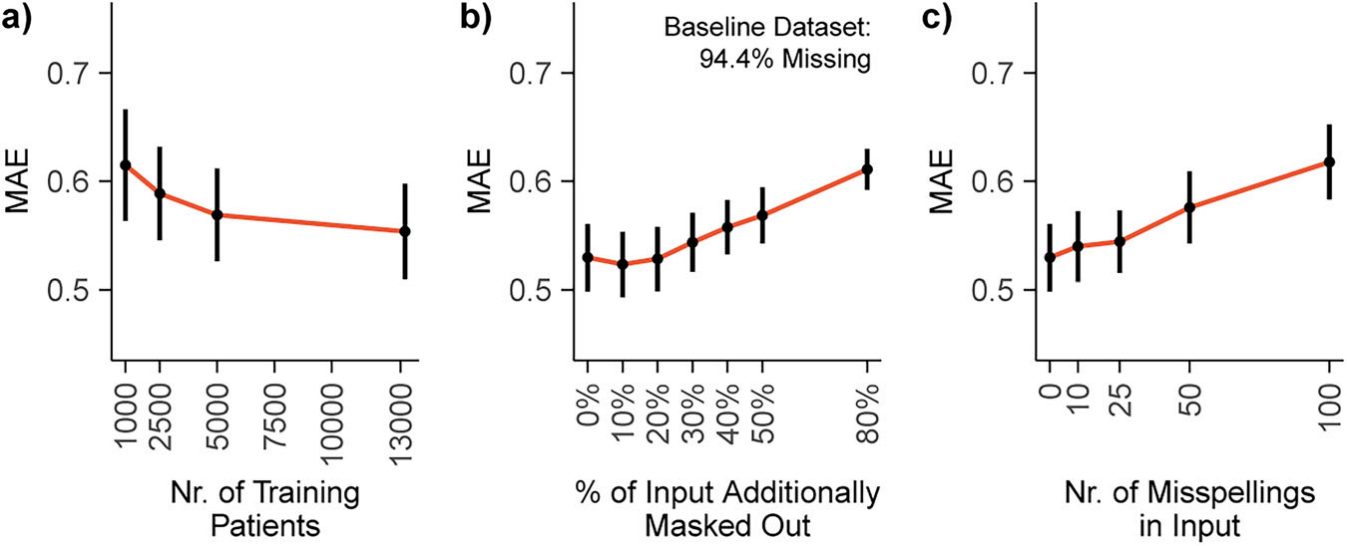
DT-GPT is robust to common RWD issues in the long-term NSCLC dataset. **a** Mean absolute error (MAE) according to the number of patients in the training set. Assessing impact on MAE based on (**b**) added missingness, on top of the baseline 94.4% missingness of the NSCLC dataset, and **c** injected misspellings in the input.

DT-GPT is flexible and robust to common practical data challenges, exhibiting desired properties in a variety of ablation studies, here exemplified on the average performance on all six clinical variables of the NSCLC dataset. First, DT-GPT performance was competitive with baselines after training with data corresponding to 5000 patients and it further improved with the number of patients in the training dataset (Fig. 4a; Table 1), and consistent in further subsampling ablation studies (Supplementary Table 20). Additionally, DT-GPT could handle increased input missingness, with performance degradation only showing after more than 20% of the input was randomly masked, on top of the 94.4% initial missingness of the NSCLC dataset (Fig. 4b). Thirdly, DT-GPT was stable to misspellings in the input, only significantly degrading in performance after 25 misspellings per patient sample (Fig. 4c). We note that misspellings cannot be handled by most established machine learning methods and either require completely dropping or manual curation of the data.

### DT-GPT enables prediction insights and zero-shot forecasting

**Fig. 5:**
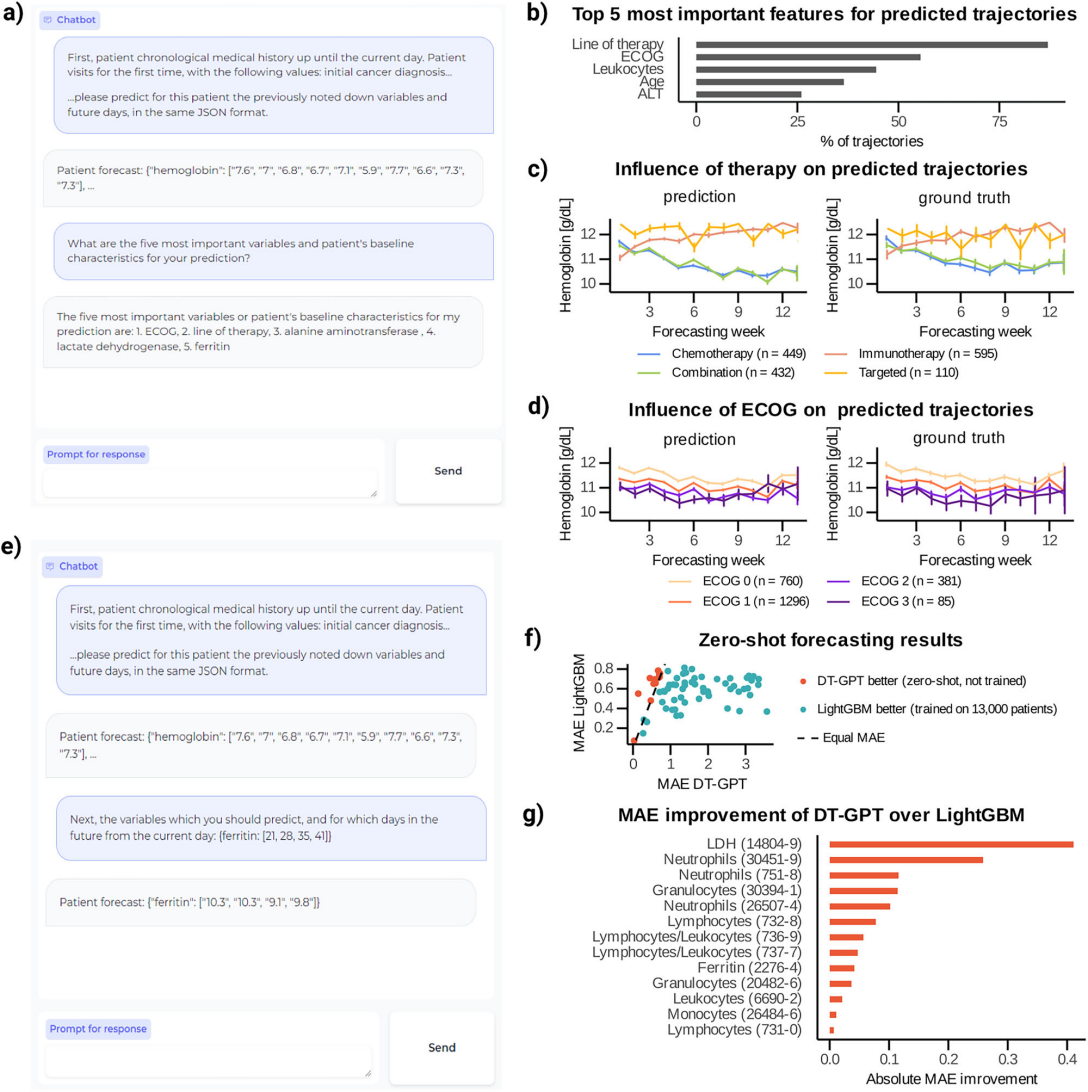
DT-GPT preserves its conversational ability after the fine-tuning, allowing inquiring into prediction rationale and zero-shot forecasting. **a** Example of a chatbot interaction providing explanations for predictions. **b** Five most important variables for predicting all variables derived from forecasting test patient samples with 10 predicted trajectories each. **c** The most important variable, therapy, influences predicted hemoglobin trajectories, with (**d**) the corresponding ground truth. Here, the lines show average trajectories and the error bars correspond to the standard error. **e** The second most important variable, ECOG, influences predicted hemoglobin trajectories, and **f** showing the corresponding ground truth. Lines represent average trajectories and the error bars correspond to the standard error. **g** Example of a chatbot interaction for forecasting a variable not previously trained on. **h** We train 69 separate LightGBM models on other variables, whilst the single DT-GPT model receives no further training, resulting in DT-GPT outperforming LightGBM models on 13 out of 69 non-target variables. **i** DT-GPT is superior for variables more biologically related to the target variables used during fine tuning, with the respective LOINC codes depicted in parentheses. ECOG Eastern Cooperative Oncology Group performance status scale, LDH lactate dehydrogenase, ALT alanine aminotransferase).

DT-GPT retains its conversational capability post-fine-tuning for the forecasting task, facilitating user interaction and enabling the inquiries into the reasoning behind predictions. For each patient sample, 10 predicted trajectories were generated, accompanied by a set of explanatory variables elucidating these predictions (Fig. 5a). We extracted explanatory variables from 25,575 out of 27,730 chatbot responses. The most influential variables were therapy, ECOG status and leukocyte count (Fig. 5b; Supplementary Table 21; Supplementary Figs. 4–9; Supplementary Note 3).

Therapy emerged as a key determinant of hemoglobin dynamics, aligning with existing literature^38,39^. Patients receiving immunotherapy and targeted therapy generally exhibited higher hemoglobin levels over time compared to those undergoing chemotherapy or combination therapies (i.e., chemotherapy and immunotherapy), where hemoglobin levels tended to decline due to the chemotherapy-induced bone marrow suppression (Fig. 5c; Supplementary Fig. 4)^40^.

ECOG status also played a significant role in shaping hemoglobin trajectories. The last recorded ECOG value in a patient’s medical history was predictive of future hemoglobin levels (Fig. 5d), with lower ECOG values—indicative of fewer performance restrictions—correlating with higher hemoglobin levels over time, consistent with prior research^41,42^. Additionally, age has been widely recognized as an important prognostic factor^43^. Notably, these findings are also reflected in the original data, reinforcing the validity of DT-GPT’s predictions (Supplementary Fig. 9).

DT-GPT enables zero-shot forecasting of non-target clinical variables, expanding its applicability beyond fine-tuned predictions. It can forecast 69 non-target clinical variables that are recorded in patient medical histories but were not explicitly included during model fine tuning. In our experiments, we forecasted each non-target variable separately (Fig. 5e) and extracted 81,004 trajectories from 81,918 forecasting results.

To benchmark DT-GPT’s zero-shot performance, we compared it against a traditional machine learning approach. We extensively trained 69 LightGBM models, each using data from over 13,000 patients for individual target variables, and compared their performance to a single DT-GPT model that received no such additional training (i.e., zero-shot setting) and therefore was at a disadvantage. LightGBM was therefore anticipated to perform better than the zero-shot DT-GPT model.

Surprisingly, zero-shot DT-GPT outperformed LightGBM on 13 out of 69 non-target variables (Fig. 5f. The variables with improved performance can be described as closely related to the target variables (Fig. 5g). For instance, *segmented neutrophils*, *band form neutrophils* and *neutrophils by automated count* have different LOINC codes from the trained variable (30451-9, 26507-4, 751-8, respectively), but these measurements were functionally related to the target variable *neutrophils* (LOINC 26499-4). A table containing scaled MAE values for DT-GPT and the LightGBM baseline is provided in Supplementary Table 22.

We identified that DT-GPT performs better in zero-shot predictions for variables highly correlated with the fine-tuned targets. Specifically, 11 of 13 non-target variables for which DT-GPT demonstrates equal or superior performance compared to LightGBM, exhibit a strong Spearman correlation coefficient ( | ρ | > 0.7) with at least one fine-tuned target variable (Supplementary Fig. 10). For the remaining well-performing zero-shot targets without strong correlations, feature importance analysis and relevant literature suggest that DT-GPT may capture clinically meaningful relationships, such as the ferritin-to-hemoglobin ratio and components of the Albumin-Bilirubin (ALBI) score in NSCLC patients (Supplementary Fig. 11; Supplementary Table 23)^44–46^.

## Discussion

Our main finding is that a simple yet effective method allows training LLMs on EHRs and study data to generate detailed patient trajectories that preserve inter-variable correlations. This method achieves state-of-the-art performance in clinical forecasting, while closely reproducing the distribution of original data and outperforming baselines in predicting clinically meaningful events in the trajectory. This highlights the potential of using LLMs as a digital twin platform that can mimic individual patients, with applications such as treatment selection and clinical trial support.

Building on past LLM research in general forecasting, DT-GPT outperforms existing baselines^20,21^ in NSCLC, ICU and Alzheimer’ s disease datasets. These findings align with recent LLM forecasting developments, demonstrating that clinically-specific adjustments enable accurate predictions^18,19^. Further analysis of several existing LLM forecasting approaches reveals that channel dependent modeling is a crucial aspect for patient trajectories, with DT-GPT showing that even a simple approach here can be highly effective. Notably, fine-tuning remains necessary for optimal performance, as demonstrated by the lower accuracy of non-fine-tuned LLMs, even when benchmarked against significantly larger models. Additionally, DT-GPT’s generative nature allows for multiple trajectory simulations per patient, offering insights into possible patient scenarios, cohort simulations, and uncertainty estimates. Finally, while all models were optimized for the forecasting task only, DT-GPT consistently outperformed baselines in classification tasks in detecting clinically relevant events by achieving best or second-best performance.

The positive performance of LLMs for patient forecasting may stem from parallels between natural language and biomedical data, such as non-random missingness. For example, a doctor might skip measuring blood pressure if a patient appears healthy, indicating information by omission. Natural language implicitly handles such ambiguity; unspoken words can still convey meaning or none at all. Recent advancements suggest that LLMs can capture these complex relationships^47^.

DT-GPT addresses EHR challenges including noise, sparsity, and lack of data normalization^16^. Unlike most established machine learning models that require data normalization and imputation, DT-GPT operates without these requirements. Here, we demonstrated its robustness to sparsity, misspellings, and noisy medical data often encountered in real-world datasets. Moreover, EHR data often contain mixed data encodings; for instance, drug information may vary in encoding, such as the dosage used or noted only as “administered”, both of which DT-GPT handles without additional preprocessing. Overall, DT-GPT simplifies and streamlines data preparation, thus enabling faster deployment across diverse datasets.

DT-GPT can be inquired about the rationale of predictions, which increases the interpretability of the model. This capability helps bridge the gap between medical expert and model, enabling the exploration of prediction rationales and alternative patient scenarios efficiently. We believe that this advancement could enhance human-computer interaction with AI predictions and may positively affect clinical practices in the near future.

DT-GPT enables zero-shot predictions, demonstrating its ability to forecast variables not explicitly included in its fine-tuning phase by learning their dynamics and adapting to novel tasks. Remarkably, zero-shot DT-GPT outperforms a supervised, fully-trained machine learning model on a subset of clinical variables, highlighting the pioneering potential of LLM-based approaches in RWD forecasting.

Applying the preliminary interpretability approach also on the zero shot variables, we hypothesize that the model is potentially able to capture latent clinical knowledge, such as the importance of the ferritin-to-hemoglobin ratio and parts of the Albumin-Bilirubin (ALBI) score, both which are emerging prognostic biomarkers in NSCLC^45,46^. It is important to note that the underlying BioMistral 7B model was trained on a vast amount of biomedical databases and publications. Therefore, these are preliminary hypotheses that require extensive investigation and validation from clinical experts.

DT-GPT shows promise for clinical trajectory forecasting, with strong performance on standard metrics (e.g., MAE) and robust modeling of temporal dependencies. It effectively detects moderate abnormalities such as anemia, tracks inflammation-related trends, and predicts progression markers such as elevated LDH. However, performance declines for specific acute events—e.g., severe hemoglobin drops or high leukocyte counts—highlighting the challenge of forecasting low-prevalence, high-variance outcomes. Future improvements will require methods that enhance sensitivity to high-risk events, such as tailored loss functions, anomaly detection, and integration of unstructured clinical data.

A challenge of LLM-based models is the restricted number of simultaneously forecasted variables. The current constraint on the number of forecasted variables is due to the limited sequence length of both input and output of the LLMs used in fine-tuning. Advances in extending the context length will enable modeling of additional patient variables, such as by using larger, more advanced models such as Qwen3-32B as the base model. Furthermore, we anticipate that transitioning from zero-shot to few-shot learning, where the model receives further training on a small subset of data, would enable a wider span of forecasted variables and extend DT-GPT’s applicability to broader clinical challenges.

Future work can also take inspiration from developments in LLM-based forecasting. Specifically, ideas such as patching and prompt-as-a-prefix from Time-LLM^22^, as well as normalization and generation of continuous likelihoods from LLMTime^21^, can be adapted for clinical use, further improving forecasting performance. Additionally, even though DT-GPT was able to capture the clinically relevant events better than other models, performance can still be improved to increase clinical relevance, therefore we consider the optimization of the classification performance to be an important direction of future work. Related to this, future research should also focus on developing disease-specific forecasting metrics that correlate well with clinical utility.

Another established shortcoming of LLM-based models is their tendency to hallucinate, as well as recreating the biases from the underlying data. In our case, the hallucination could be reflected in explainability results not necessarily providing true answers. This is a critical aspect for the medical domain, and we believe that a human-in-the-loop setup will be required, together with advanced training of clinicians on the use of LLM outputs. Regarding model biases, it is well established that models recreate the biases from the underlying data, which is especially pronounced in minority populations^48^. To overcome the bias issues, methodological work, training of users, as well as the gathering of large scale, diverse clinical datasets, is needed.

Finally, we observe that high error predictions often occur due to the high variance between the multiple generated trajectories of each patient sample, with the mean aggregation into the final prediction not capturing key dynamics. It is thus an open challenge to develop improved aggregation methods, for example by using a second LLM as an arbiter or by having a human expert select the most realistic trajectory.

In conclusion, DT-GPT highlights the utility of using LLMs as a digital twin forecasting platform, enabling state-of-the-art and stable predictions, exploratory interpretability via a natural-language interface, and forecasting of patient variables not used in fine-tuning. Whilst further advancements are needed for wide-scale deployment, DT-GPT exhibits digital twin behaviors, potentially reproducing many aspects of the patients it represents, and surpassing traditional AI methods optimized for individual variables. We believe that through further method development and extensive validation, patient-level digital twins will impact clinical trials by supporting biomarker exploration, trial design, and interim analysis. Additionally, future digital twins will assist doctors in treatment selection and patient monitoring. Overall, we envision LLM-powered digital twins becoming integral to healthcare systems.

## Methods

**Fig. 6:**
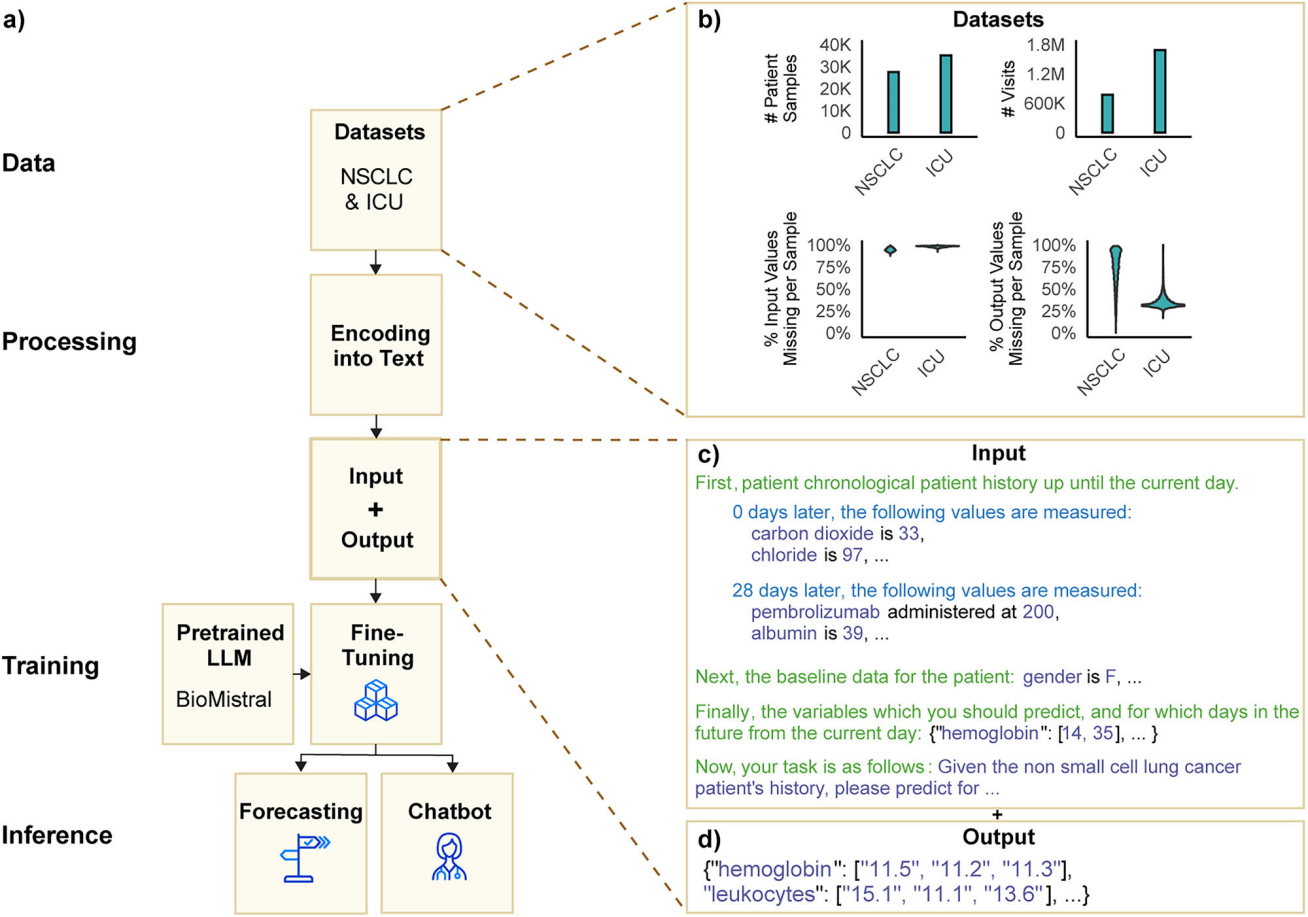
The DT-GPT framework transforms EHRs into text and subsequently fine-tunes an LLM on this data. **a** Overview of the pipeline: datasets are split and encoded into input/output text based on landmark timepoints, then used to fine-tune an LLM, here BioMistral. The model output is evaluated for trajectory forecasting whilst zero-shot predictions and variable importances are explored via a chat interface. **b** Sample size, visit frequency, and sparsity of the Alzheimer’s disease (AD), non-small cell lung cancer (NSCLC), intensive care unit (ICU) datasets. **c** Input and **d** output encoded examples, emphasizing the chronological encoding of observations.

DT-GPT is a method that employs pre-trained LLMs fine-tuned on clinical data (Fig. 6a). Notably, this method is agnostic regarding the underlying LLM and can be applied without architectural changes to any general-purpose or specialized text-focused LLM. We trained and evaluated DT-GPT for forecasting patients’ laboratory values across three independent datasets, i.e., non-small cell lung cancer (NSCLC), intensive care unit (ICU), and Alzheimer’s disease patients.

### NSCLC dataset

For the US-based NSCLC dataset, we used the nationwide Flatiron Health EHR-derived de-identified database. The data are de-identified and subject to obligations to prevent re-identification and protect patient confidentiality. The Flatiron Health database is a longitudinal database, comprising de-identified patient-level structured and unstructured data, curated via technology-enabled abstraction^49,50^. During the study period, the de-identified data originated from approximately 280 cancer clinics ( ~ 800 sites of care).

The study included 16,496 patients diagnosed with NSCLC from 01 January 1991 to 06 July 2023. The majority of patients in the database originate from community oncology settings; relative community/academic proportions may vary depending on the study cohort. Patients with a birth year of 1938 or earlier may have an adjusted birth year in Flatiron Health datasets due to patient de-identification requirements. To harmonize the data, we aggregated all values in a week based on the last observed value.

We focused on the 50 most common diagnoses and 80 most common laboratory measurements, complemented by the Eastern Cooperative Oncology Group (ECOG) score, metastases, vitals, drug administrations, response, and mortality variables totaling 773,607 patient-days across 320 variables.

For every NSCLC patient, we divided their trajectory into input and output segments based on the start date of each line of therapy to create each patient sample. All variables up to the start date were considered input data. The objective was to predict the weekly values up to 13 weeks after the start date of the following variables and their respective LOINC codes: hemoglobin (718-7), leukocytes (26464-8), lymphocytes/leukocytes (26478-8), lymphocytes (26474-7), neutrophils (26499-4) and lactate dehydrogenase (2532-0). These variables were selected due to their frequent measurement and relevance in reflecting key characteristics of NSCLC treatment response (Supplementary Tables 1, 2).

### ICU dataset

To demonstrate the generalizability of DT-GPT, we analyzed ICU trajectories from the publicly-accessible Medical Information Mart for Intensive Care IV (MIMIC-IV) dataset^25^. We employed an established processing pipeline, resulting in 300 input variables across 1,686,288 time points from 35,131 patients^51^.

Here, the objective was to predict a patient’s future hourly lab variables given their first 24 h in the ICU. Specifically, the patient history was considered as the first 24 h for all variables, and the task was to forecast the future 24 hourly values for the following variables: O2 saturation pulse oximetry, respiratory rate and magnesium. These variables were selected due to having the highest temporal variability, thus making the forecasting task more challenging, and the fact that at least 50% of patients had at least one measurement for each, highlighting their widespread clinical usage (Supplementary Tables 1, 3, 4). These criteria not only increased the forecasting challenge, but also ensured wide representation across the patient population.

### Alzheimer’s disease dataset

To further demonstrate the generalizability of DT-GPT, we ran DT-GPT and the baseline models on the Alzheimer’s disease dataset, based on the Alzheimer’s Disease Neuroimaging Initiative (ADNI) database (adni.loni.usc.edu). The ADNI was launched in 2003 as a public-private partnership, led by Principal Investigator Michael W. Weiner, MD. The primary goal of ADNI has been to test whether serial magnetic resonance imaging (MRI), positron emission tomography (PET), other biological markers, and clinical and neuropsychological assessment can be combined to measure the progression of mild cognitive impairment (MCI) and early Alzheimer’s disease (AD).

We preprocessed the dataset, including 1140 patients. The task was to predict the 24 month trajectory of three cognitive variables, given the baseline measurements of the patients. Specifically, the variables were Mini Mental State Examination (MMSE), Clinical Dementia Rating sum of boxes (CDR-SB) and Alzheimer’s Disease Assessment Scale (ADAS11), which are key indicators of cognitive decline commonly measured in Alzheimer’s disease patients (Supplementary Tables 1, 5).

### Data splitting and filtering

The NSCLC and ICU datasets were split at the patient level into 80% training, 10% validation, and 10% test set. The splitting was performed randomly for the ICU dataset, whilst stratified by group stage, smoking status, number of observations per visit and number of visits with drug administrations to ensure a balanced evaluation. The Alzheimer’s disease dataset was randomly split into 80% training and 20% test, selected due to the small sample size, with all hyperparameters determined via a further splitting on the training set. Thus, each set comprised disjoint sets of patients to avoid data leakage. The test sets were solely used for final evaluation and to assess the model’s generalizability (Fig. 6b).

We applied a two-step outlier filtering procedure on all datasets: all target values below or above three standard deviations were filtered out first, then we calculated new standard deviation values on the filtered dataset and clipped target values below and above those values. This approach ensured that the noise present in the data was removed, while some of the outliers were replaced with reasonable low or high values to maintain the biological signal. The data for all of the baselines excluding DT-GPT were then also standardized using z-scores.

### Encoding

We encoded patient trajectories by using templates that converted medical histories based on EHRs into a text format compatible with LLMs, as proposed by Xue et al.^19^ and Liu et al.^19,20^ (Fig. 6c, d; Supplementary Note 4). The input template is structured into four components: (1) patient history, (2) demographic data, (3) forecast dates and (4) prompt. The patient history contains a chronological description of patient visits, requiring no data imputation for missing variables. The output trajectories were also encoded using templates, containing only the relevant output variables for the forecasted time points. We utilized a manually developed template for input encoding and JSON-format encoding for the output (Supplementary Fig. 12).

### LLMs and fine-tuning

We utilized the biomedical LLM BioMistral 7B DARE, since it is provided with an open source license and based on a recognized LLM^33^. Furthermore, BioMistral is instruction tuned and through its biomedical specialization incorporates compressed representations of vast amounts of biomedical knowledge. We further fine tuned this LLM using the standard cross entropy loss, masked so that the gradient was only computed on the output text. We performed 30 predictions for each patient sample during evaluation, then took the mean for each time point as the final prediction^21,52^. All hyperparameters of DT-GPT used fine-tuning (Supplementary Note 5) and are compared to baseline models (Supplementary Note 6).

### Handling of missing and noise data

We investigated the ability of DT-GPT as a LLM-based model to handle missing data and misspelling in the input prompts. For the missing data study, we randomly masked between 0 and 80% of data, in addition to the already missing data in a dataset. Evaluation of the effect of missingness was performed on a randomly sampled 200 patients from the test set, which can potentially lead to higher variance in the results, but allowed for a more extensive exploration.

For the noise study, we introduce a misspelling algorithm. This algorithm randomly performs either perturbation, insertion, deletion, or replacement, using all ASCII letters & digits, applied to the entire input text. This includes dates, variable names, values, baseline information, and prompts. One operation is considered one misspelling.

For the evaluation of the effects of RWD missingness and noise we randomly sampled 200 patients of the test set, which can potentially lead to higher variance in the results, but allowed for a more extensive exploration.

### Chatbot and zero-shot learning

We employed the DT-GPT model to run a chatbot based on patient histories for prediction explanation and zero-shot forecasting. For this, first we used DT-GPT to generate forecasting results from patient history and, consecutively, added a task-specific prompt surrounded by the respective instruction-indication tokens to the DT-GPT chat history for receiving a response. For prediction explanation, the prompt asked for the most important variables influencing the predicted trajectory. For zero-shot forecasting, the prompt specified the output format and days to predict new clinical variables that were not subject to optimization during training. Example prompts and chatbot interactions for both tasks are provided in Supplementary Note 7 and Fig. 5a, e.

### Forecasting evaluation

Forecasting metrics, i.e. Eqs. (1)–(5), are designed to quantify the disparity between predicted and observed numeric values, providing an objective measure of the model’s predictive accuracy (Supplementary Note 8). Let ${{v}_{t}}^{(i)}$ be an observed (non-missing) value of clinical variable $v$ for a subject $i$, $i=1,\cdots ,n$, where $n$ is the total number of subjects, and time step $t$, $t=1,\cdots ,{T}_{i}$, where ${T}_{i}$ is the total number of time steps for the subject $i$. Let baseline value ${v}_{0}^{(i)}$ be the baseline value at time step ${t}_{0}$, $t=0$. We denote predicted values as ${\hat{v}}_{t}^{(i)}$. The forecasting metrics used are mean absolute error (MAE), scaled MAE, mean absolute scaled error (MASE), symmetric mean absolute percentage error (SMAPE) and Spearman correlation coefficient defined as follows:

1 $${MAE}=\frac{1}{n}\mathop{\sum }\limits_{i=1}^{n}\frac{1}{{T}_{i}}\mathop{\sum }\limits_{t=1}^{{T}_{i}}|{v}_{t}^{(i)}-{\hat{v}}_{t}^{(i)}|$$

2 $${scaled\; MAE}=\frac{{MAE}}{\sigma }$$

where $\sigma$ is the standard deviation of the clinical variable after outlier filtering;

3 $${MASE}=\frac{{MAE}}{\,\frac{1}{n}{\sum }_{i=1}^{n}\frac{1}{{T}_{i}}{\sum }_{t=1}^{{T}_{i}}|{v}_{t}^{(i)}-{v}_{0}^{(i)}|}$$

4 $${SMAPE}=\frac{200}{n}\mathop{\sum }\limits_{i=1}^{n}\frac{1}{{T}_{i}}\mathop{\sum }\limits_{t=1}^{{T}_{i}}\frac{{|{{v}_{t}}^{(i)}\,-{{\,\hat{v}}_{t}}^{(i)}|}}{|{v}_{t}^{(i)}|+|{\hat{v}}_{t}^{(i)}|}{\mathbb{1}_{\{|v_t^{(i)}| + |\hat{v}_t^{(i)}| \neq 0\}}}$$

where ${\mathbb{1}}$ is the indicator function to avoid division by 0;

5 $$Spearman\,\rho =\frac{{\sum }_{i=1}^{n}\,\frac{1}{{T}_{i}}{\sum }_{t=1}^{{T}_{i}}\,(R[{v}_{t}^{(i)}]-\underline{R[v]})(R[{\widehat{v}}_{t}^{(i)}]-\underline{R[\widehat{v}]})}{\sqrt{{\sum }_{i=1}^{n}\,\frac{1}{{T}_{i}}{\sum }_{t=1}^{{T}_{i}}{(R[{v}_{t}^{(i)}]-\underline{R[v]})^{2}}\,{\sum }_{i=1}^{n}\frac{1}{{T}_{i}}\,{\sum }_{t=1}^{{T}_{i}}\,(R[{\widehat{v}}_{t}^{(i)}]-\underline{R[\widehat{v}]})^{2}}}$$

where $R[.]$ is a rank function, ordering values from lowest to the highest, whereby, for the data points with the same value, their average rank is assigned, and $\underline{R[v]}=\frac{1}{n}{\sum }_{i=1}^{n}\,\frac{1}{{T}_{i}}{\sum }_{t=1}^{{T}_{i}}\,R[{v}_{t}^{(i)}]$ and $\underline{R[\widehat{v}]}=\frac{1}{n}\,{\sum }_{i=1}^{n}\,\frac{1}{{T}_{i}}\,{\sum }_{t=1}^{{T}_{i}}\,R[{\widehat{v}}_{t}^{(i)}]$ are the mean ranks of actual and predicted values, respectively.

We chose scaled MAE, i.e., Eq. (2), as our primary metric as it allows comparison across all variables, and hence can be used to benchmark different models on all datasets.

#### Classification evaluation

Classification metrics assess the model’s clinical utility to capture events, such as abrupt changes in clinical variables indicative of acute conditions (e.g., sudden drops or increases) or prolonged trends in variable changes that are characteristic of a chronic condition (e.g., gradual increases or decreases over extended periods). Below, we provide detailed definitions of the metrics employed in our evaluation. An interpretation of introduced metrics is provided in Supplementary Note 8.

First, we assess the model’s ability to detect values outside the normal range of clinical variables. Let $[{v}_{\min \,},\,{v}_{\max }]$ be the reference interval for the clinical variable $v$. We label the observed variable value ${v}_{t}^{(i)}$ as “low” if ${v}_{t}^{(i)} < {v}_{\min }$, as “high” if ${v}_{t}^{(i)} > {v}_{\max }$ and as “normal” if ${v}_{\min } < {v}_{t}^{(i)} < {v}_{\max }$. We define ${v}_{t}^{(i)}$ as “not low” if it is “normal” or “high”, as “not high” if it is “normal” or “low”, and as “not normal” if it is “low” or “high”. Analogously, we label each predicted variable value ${\hat{v}}_{t}^{(i)}$. With this, we are in the classification task settings.

For the binary classification tasks “low” versus “not low”, “high” versus “not high”, and “normal” versus “not normal”, we calculate area under the receiver operating characteristic curve (AUC ROC) and denote it as ${AU}{C}_{{low}}$, ${AU}{C}_{{high}}$ and ${AU}{C}_{{normal}}$, respectively. For the multiclass classification task “low” versus “normal” versus “high”, we calculate weighted AUC ROC, denoted by AUC weighted (Eq. (6)), that is given by

6 $$\text{AUC}_{\text{weighted}} = \frac{(\text{AUC}_{\text{low}} \times \#\text{low}) + (\text{AUC}_{\text{normal}} \times \#\text{normal}) + (\text{AUC}_{\text{high}} \times \#\text{high})}{\#\text{low} + \#\text{normal} + \#\text{high}}$$

where ${\#low}$, ${\#normal}$ and ${\#high}$, correspond to the number of observed variables values ${{v}_{t}}^{(i)}$ labeled as “low”, “normal” and “high” respectively. Weighted aggregation accounts for the class imbalance, whereby most of the variable values fall within the reference range and are labeled as “normal”.

We evaluated the model’s trend forecasting performance by analyzing its predicted value trajectories over a specified time interval $s$. Within these forecasts, a predicted value ${v}_{t}^{(i)}$ was classified as ‘decreasing trend’ if ${v}_{t+1}^{(i)} < {v}_{t}^{(i)}$ or as an ‘increasing trend’ if ${v}_{t+1}^{(i)} > {v}_{t}^{(i)}$. For a trend to be classified at time $t$, the direction of change between consecutive predicted values had to be consistent throughout the entire preceding lookback window. Specifically, ${v}_{t}^{(i)}$ was classified as ‘decreasing trend’ only if ${v}_{k+1}^{(i)} < {v}_{k}^{(i)}$ for all time steps $k$ within the interval $[{time}(t)-s,{time}(t)]$, and ‘increasing trend’ only if ${v}_{k+1}^{(i)} > {v}_{k}^{(i)}$ for all $k$ in that same interval. Here, ${time}(t)$ represents the time since the last input measurement. Ground truth trends were derived similarly from observed data. We then assessed the model’s classification of these trends in its forecasts using two binary classification tasks: ‘decreasing’ versus ‘not decreasing’, and ‘increasing’ versus ‘not increasing’. Performance was quantified by calculating the area under the receiver operating characteristic curve (AUC) based on the forecasted values, yielding ${AU}{C}_{{trend}\downarrow }$ and ${AU}{C}_{{trend}\uparrow }$. Forecasted values were excluded from this analysis if ${time}(t) < s$ to ensure a complete lookback window was available. We provide an example and illustration in Supplementary Fig. 13.

We performed the classification evaluation only on the NSCLC data. For this, we used parameters for the reference ranges $[{v}_{\min },\,{v}_{\max }]$ as found in the literature. For hemoglobin [g/dL], we set [14, 18] and [12, 16] for male and female patients^53^, respectively. We set [4.5, 11.0] for leukocytes [10^9^/L]^54^, [20, 40] for leukocytes/lymphocytes [%]^54^, [1.0, 4.0] for lymphocytes [10^9^/L]^55^, [1.8, 7.5] for neutrophils [10^9^/L]^55^ and [122, 222] for lactate dehydrogenase [U/L]^36^.

We further address the model ability to detect a significant drop in hemoglobin associated with a bleeding by calculating ${AU}{C}_{{low}}$ with ${v}_{\min }$ = 7.5. As for the trend detection, we consider time intervals of 3 weeks and set $s=21$ days for all NSCLC variables. This time period is clinically relevant to capture the increasing or decreasing dynamics of a clinical variable.

## Supplementary information

Supplementary Information

## Data Availability

The Flatiron Health data that support the findings of this study were originated by and are the property of Flatiron Health, Inc., which has restrictions prohibiting the authors from making the data set publicly available. Requests for data sharing by license or by permission for the specific purpose of replicating results in this manuscript can be submitted to PublicationsDataAccess@flatiron.com. The Medical Information Mart for Intensive Care IV (MIMIC-IV) is available online upon request under https://physionet.org/content/mimiciv. The Alzheimer’s Disease Neuroimaging Initiative (ADNI) dataset is available online upon request under https://adni.loni.usc.edu/data-samples/adni-data/.

## Code availability

The code is available at https://github.com/MendenLab/DT-GPT, including all package and Python versions, as well as license information. Specific parameters used to generate and analyze the datasets presented in this manuscript are detailed in the repository’s README file and relevant configuration files.
